# Antibiotics for acute watery or persistent with or without bloody diarrhoea in children: A systematic review and meta-analysis

**DOI:** 10.7189/jogh.14.04211

**Published:** 2024-12-06

**Authors:** Syeda Kanza Naqvi, Mustafa Bin Ali Zubairi, Ayesha Arshad Ali, Ashraf Sharif, Rehana Abdus Salam, Zain Hasnain, Sajid Soofi, Shabina Ariff, Yasir Bin Nisar, Jai K Das

**Affiliations:** 1 Institute for Global Health and Development, Aga Khan University, Karachi, Pakistan; 2 University Library, Aga Khan University, Karachi, Pakistan; 3 The Daffodil Centre, The University of Sydney, a joint venture with Cancer Council NSW, Sydney, New South Wales, Australia; 4 Centre of Excellence in Women and Child Health, Aga Khan University, Karachi, Pakistan; 5 Department of Pediatrics & Child Health, Aga Khan University, Karachi, Pakistan; 6 Department of Maternal, Newborn, Child, and Adolescent Health and Ageing, World Health Organization, Geneva, Switzerland; 7 Division of Women and Child Health, Aga Khan University, Karachi, Pakistan

## Abstract

**Background:**

The use of antibiotics in the treatment of acute and persistent watery diarrhoea has long been a subject of contention. While the advantages of using antimicrobials are acknowledged, concerns remain regarding potential adverse effects and antibiotic resistance. Therefore, we conducted a systematic review and meta-analysis to assess the efficacy of antibiotics compared to placebos for the treatment of diarrhoea.

**Methods:**

We searched PubMed, CINAHL, the Cochrane Library, ClinicalTrials.gov, the World Health Organization (WHO) International Clinical Trials Registry Platform, and Scopus from inception until 20 July 2023 for studies published after the year 2000 assessing antibiotics vs placebo in acute and persistent diarrhoea and/or blood in stools in children less than 10 years of age. We conducted a meta-analysis for the included studies, assessed them using the Risk of Bias 2 tool, and evaluated their quality of evidence through the Grading of Recommendations, Assessment, Development, and Evaluations (GRADE) framework. This review was commissioned by WHO for revision of their guidelines for childhood diarrhoea management.

**Results:**

We included five randomised controlled trials (RCTs) for acute watery diarrhoea and no study for bloody diarrhoea. Our findings suggest that there is a significant increase in clinical cure (risk ratio (RR) = 2.28; 95% confidence interval (CI) = 1.52, 3.41; low certainty evidence) and parasitological cure (RR = 2.86; 95% CI = 1.72 to 4.74; low certainty evidence) among children with acute watery diarrhoea in the antibiotic group when compared to the placebo group. The duration of diarrhoea (in hours) was significantly reduced (mean difference = −24.90; 95% CI = −34.09, −15.71; low certainty evidence) in the intervention group, while the effect on all-cause mortality (RR = 0.71; 95% CI = 0.40, 1.27; moderate certainty evidence) and the need for intravenous fluid infusion (RR = 0.50; 95% CI = 0.05, 5.17; very low certainty evidence) were comparable between the two groups.

**Conclusions:**

In children under 10 years of age suffering from acute watery or persistent diarrhoea, antibiotics led to an apparent increase in cure rates. However, considering the low certainty of evidence, low number of studies with small sample sizes, and the fact that most studies were conducted in a single country, further investigation and cautious interpretation are warranted, as is a large multi-country RCT that would allow for firmer conclusions.

**Registration:**

PROSPERO: CRD42023447133.

The Sustainable Development Goal 3 (SDG 3) calls for a reduction in under-five mortality to 25 per 1000 live births by 2030 [1], to which diarrhoea still contributes significantly, despite a more than 30% reduction in diarrhoea-specific mortality over the last 15 years [2]. Acute diarrhoea, in fact, is still the second largest cause of morbidity and mortality globally [2], especially in low- and middle- income countries (LMICs) of sub-Saharan Africa and South Asia [3]. Diarrhoea accounts for 9% of all mortality in children under five, translating to over 1300 children dying each day, or about 484 000 children in the year 2019 [4].

Diarrhoea can be classified as acute watery diarrhoea, dysentery, persistent diarrhoea, and chronic diarrhoea, each having its distinct aetiology and management approach [5,6]. The risk factors for the condition include contaminated water, poor health, malnutrition, high-level contact with pathogens, and sub-optimal breastfeeding [7,8]. Acute watery diarrhoea is non-inflammatory in nature and manifests without red or white blood cells in faeces [9]; it is commonly caused by viral pathogens, including rotavirus. Dysentery, in turn, is mainly caused by viruses, toxin-producing bacteria, or parasites [10,11], and is characterised by acute blood in stools necessitating the use of antimicrobials.

Despite the World Health Organization (WHO) guidelines suggesting against the routine use of antimicrobials for acute watery diarrhoea, antibiotics are being widely used for treating the condition, necessitating a thorough investigation into their actual efficacy to reassess the current recommendations. This is especially true given the contrasting findings from prior research, as some studies suggested the use of antibiotics and others observed that they can lead to prolongation of the disease [12]. Considering this, we undertook a systematic review and meta-analysis to assess the efficacy of antibiotics in acute or persistent diarrhoea, with or without blood in the stool, in children under the age of 10 years. This review was commissioned by the WHO in order to generate evidence for the revision of guidelines for childhood diarrhoea.

## METHODS

### Objective

The objective of this systematic review is to assess the effectiveness of antibiotics when compared to placebo for the management of acute watery or persistent with or without bloody diarrhoea in children less than 10 years of age. The protocol for this review was registered in PROSPERO (CRD42023447133).

### Inclusion criteria

We included randomised controlled trials (RCTs) assessing the efficacy of antibiotics in the management of acute watery or persistent diarrhoea with or without blood in stools. Participants included infants and children between the ages of 0 months to 10 years (<119 months); we also included evidence from a broader age group, but inclusive of our target population (**Box 1**).

Box 1 **Inclusion and exclusion criteria**

**Inclusion criteria**
- Low-, middle-, or high-income country
- Infants and children 0 month to 10 years (<119 months) of age with acute watery or persistent diarrhoea with or without blood in stools
- Type of intervention: any antibiotic for treatment and management of acute watery or persistent diarrhoea, or any antibiotic for treatment and management of diarrhoea with blood in stools
- Comparison group: placebo
- Relevant study designs: RCT (individually or cluster)

**Exclusion criteria**
- Recruitment of adults or animals
- Studies without a comparison group
- Studies in languages other than English
- Studies in which only a proportion of participants had diarrhoea
- Studies were conducted solely on HIV patients, malnourished patients, and patients with any chronic disease.
- Studies conducted before the year 2000

### Outcomes

We selected the primary and secondary outcomes after a review of the existing literature and in discussion with the guideline development group at the WHO. Specifically, the primary outcomes were clinical cure; bacteriological/parasitological cure; treatment failure; duration of diarrhoea; and mortality. The secondary outcomes were intravenous fluid infusion and serious adverse events.

### Search strategy

We formulated the search strategy using the PICO methodology, basing it on MeSH terms and keywords, but without restrictions by outcome-related keywords to retain a broader search (**Online Supplementary Document**). We only included English-language studies from 2000 to ensure that we use the most recent evidence and to assess the common antibiotics being used.

We ran the searches in PubMed, CINAHL, the Cochrane Library, ClinicalTrials.gov, the WHO International Clinical Trials Registry Platform, and Scopus until 20 July 2023. We also searched the reference list of all included studies and relevant systematic reviews to include any missing studies. Lastly, we inputted the title of each included study into Google Scholar and screened the first 50 results.

We exported our search results into EndNote, version 20 (Clarivate, London, UK), deduplicated them, and uploaded them onto Covidence [13] for independent title/abstract and full-text screening by three researchers (KN, MZ, ZH) based on the pre-defined eligibility criteria. Disagreements were resolved through discussion or, if needed, the engagement of a third researcher (AA).

### Data extraction and management

Two researchers (KN, MZ) independently extracted data into a standardised, pilot-tested data extraction form in Microsoft Excel, version 2409 (Microsoft Corp., Redmond, Washington, USA). Two independent authors (KN, MZ) assessed the methodological quality of the included RCTs (individual or cluster) using the Cochrane Risk of Bias 2 (RoB 2) tool and give an overall risk of bias judgment (low, high, and some concerns) [14]. The RoB 2 tool assesses trials on the following domains: randomisation process, deviations from the intended interventions, missing outcome data, measurement of the outcome, and selection of the reported result. Disagreements in the assessment process were discussed and resolved either by re-evaluating the studies in question or by engaging a third author (AA or RAS). We reached out to corresponding authors for any missing information via e-mail, but we did not receive any unpublished data.

### Statistical analysis

We conducted the meta-analysis in RevMan, version 5.4.1 (Cochrane, London, UK). We used risk ratios (RRs) for dichotomous and mean differences (MDs) for the continuous outcomes, along with their 95% confidence intervals (CIs). For continuous outcomes, we converted all units to a uniform scale. Means and standard deviations (SDs) were calculated if median and interquartile ranges or CIs were provided. We then calculated mean differences using inverse variance in RevMan. If there was only one control group, the number of participants was halved in both continuous and dichotomous outcomes.

We assessed for statistical heterogeneity using τ^2^, *I*^2^, and significance of the χ^2^ test, and used random effects models where heterogeneity was present and fixed effects models where it was absent. We also performed sensitivity analysis on all outcomes to consider the impact of high risk of bias, except for outcomes where all studies had low risk of bias.

### Quality assessment (GRADE)

We used the Grading of Recommendations, Assessment, Development, and Evaluations (GRADE) criteria [15] to assess all outcomes based on the risk of bias, inconsistency, indirectness, imprecision, and publication bias and the certainty of the evidence was rated as either very low, low, moderate, or high [16]. We also generated summary of findings tables and provided the reasons for downgrading each study. The assessment was done in the GRADEpro software.

## RESULTS

**Figure 1:**
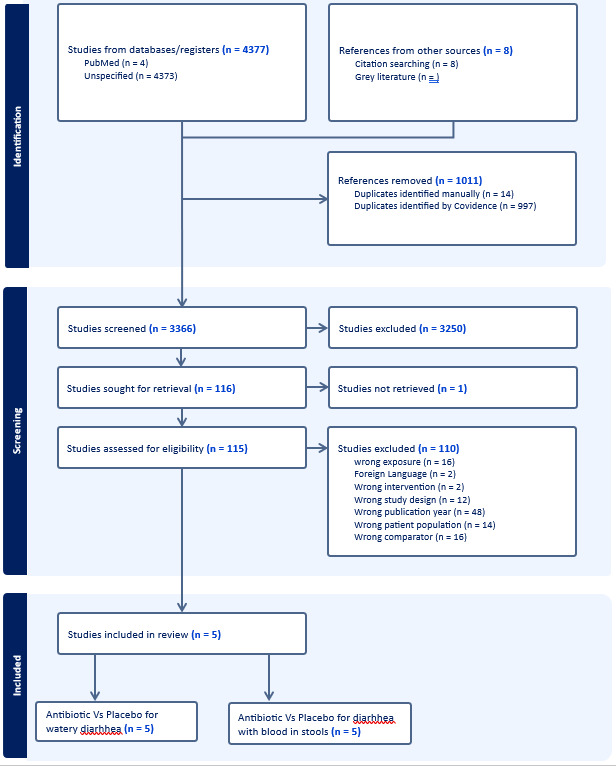
PRISMA flow diagram.

Our search retrieved 4377 records and an additional eight from grey literature. Following deduplication, 3366 remained for titles/abstracts screening. Following this stage, we reviewed 111 full texts and included five RCTs that fulfilled the inclusion criteria for acute watery and persistent diarrhoea (**Figure 1**). There were no studies comparing antibiotics vs placebo for bloody diarrhoea.

### Study characteristics

The five RCTs involved participants across various age ranges (2–23 months [17], 12–59 months [18], 1–11 years [19,20], and 4–11 years [21]) exclusively from LMICs (one in India [18], three in Egypt [19–21], and one as multi-country across Bangladesh, India, Kenya, Malawi, Mali, Pakistan, and Tanzania [17]) (**Table 1**). All studies were conducted in tertiary care settings. Four RCTs compared nitazoxanide with placebo, and one compared azithromycin with placebo.

**Table 1:** Study characteristics

Study ID	Study Design	Country	Setting	Intervention	Control	Age range	Outcomes
Rossignol et al., 2001 [19]	RCT	Egypt	Tertiary	Nitazoxanide	Placebo	1–11 y	Clinical cure, parasitological cure
Rossignol et al., 2006 [21]	RCT	Egypt	Tertiary	Nitazoxanide	Placebo	4–11 y	Mean duration of diarrhoea
Rossignol et al., 2007 [20]	RCT	Egypt	Tertiary	Nitazoxanide	Placebo	1–11 y	Clinical cure, parasitological cure
Mahapatro et al., 2017 [18]	RCT	India	Tertiary	Nitazoxanide	Placebo	12–59 mo	Mean duration of diarrhoea, need for intravenous therapy
Ahmed et al., 2021 [17]	RCT	Multi-country	Tertiary	Azithromycin	Placebo	2–23 mo	180-day all-cause mortality

mo – month, RCT – randomised controlled trial, y – year

### Primary outcomes

**Figure 2:**
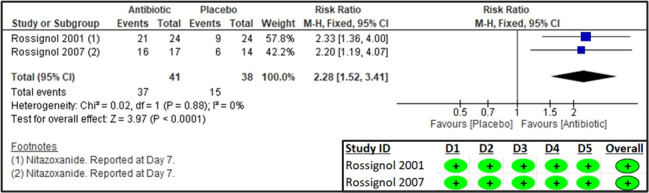
Forest plot and RoB 2 assessment for frequency of clinical cure.

**Figure 3:**
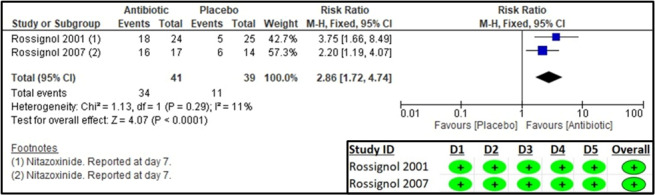
Forest plot and RoB 2 assessment for frequency of parasitological cure.

Two studies reported on clinical cure and parasitological cure [19,20]. The former was defined as patients in whom diarrhoea resolved with no symptoms, no watery stools, ≤2 soft stools, or no symptoms and no unformed stools within 48 hours, and the latter as no oocysts in post-treatment stool sample. The results suggested a significant increase in clinical cure (RR = 2.28; 95% CI = 1.52, 3.41; two studies, 79 participants, low certainty of evidence) and parasitological cure (RR = 2.86; 95% CI = 1.72, 4.74; two studies, 79 participants, very low certainty of evidence) in the antibiotic group compared to placebo. The RoB 2 assessment suggested a low risk of bias for all domains (**Figure 2**, **Figure 3**, **Table 2**).

**Table 2:** GRADE assessment table

Certainty assessment							Number of patients, n/N (%)		Effect			
**Outcome**	**Study design**	**Risk of bias**	**Inconsistency**	**Indirectness**	**Imprecision**	**Other considerations**	**Antibiotic**	**Placebo**	**RR (95% CI)**	**AR per 1000 population (95% CI)**	**Certainty**	**Importance**
Clinical cure (two studies)	RCT	Not serious	Not serious	Not serious*	Very Serious†	None	37/41 (90.2)	15/38 (39.5)‡	2.28 (1.52, 3.41)	505 (205, 951)	Low	Critical
Duration of diarrhoea (two studies)	RCT	Not serious	Not serious	Serious*	Serious†	None	43	45	-	Mean difference of 24.9 h (34.09, 15.71)	Low	Critical
All-cause mortality (one study)	RCT	Not serious	Not serious	Serious§	Serious¶	None	20/4133 (0.5)	28/4135 (0.7)	0.71 (0.40, 1.27)	2 (4, 2)	Low	Critical
Intravenous fluid therapy (one study)	RCT	Not serious	Not serious	Serious*	Very serious¶	None	1/25 (4.0)	2/25 (8.0)	0.50 (0.05, 5.17)	40 (76, 334)	Very low	Important
Parasitological cure (two studies)	RCT	Not serious	Not serious	Serious*	Very Serious†	None	34/41 (82.9)	11/39 (28.2)	2.86 (1.72, 4.74)	525 (203, 1000)	Very low	Important

AR – absolute risk, CI – confidence interval, RCT – randomised controlled trial, RR – risk ratio

*Trials conducted in populations with confirmed parasitic disease and overestimate the baseline risk.

†Small sample size. wide confidence interval.

‡3.5% baseline risk of parasitic disease in underlying population from study by Platts-Mills et al. [22].

§ABCD study has underlying population of children with watery stool with either dehydration or malnutrition and thought to overestimate a mortality benefit.

¶CI crosses null value.

**Figure 4:**
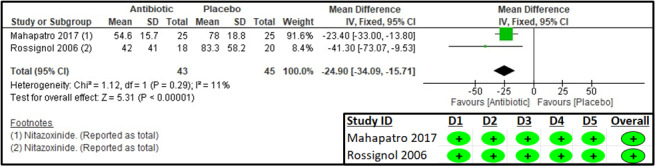
Forest plot and RoB 2 assessment for mean duration of diarrhoea (in hours).

Two studies reported the mean duration of diarrhoea in hours [18,21]. The results suggested a 24.9-hour decrease in the duration of diarrhoea in the antibiotic group when compared to the placebo group (MD = −24.90; 95% CI = −34.09, −15.71; two studies, 88 participants, low certainty evidence) (**Figure 4**). 

**Figure 5:**
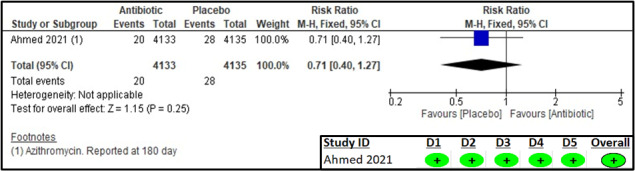
Forest plot and RoB 2 assessment for all-cause mortality.

The 180-day all-cause mortality was reported only by Ahmed et al. [17] (**Figure 5**), with comparable results between the two groups (RR = 0.71; 95% CI = 0.40, 1.27; one study, 8268 participants, low certainty of evidence) (**Table 2**).

The RoB 2 assessment for all studies in this outcome showed a low risk of bias for all domains.

### Secondary outcome

**Figure 6:**
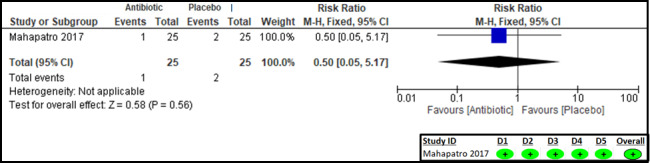
Forest plot and RoB 2 assessment for need for intravenous therapy.

Mahapatro et al. [18] reported on the need for intravenous therapy, with the results being comparable between the two groups (RR = 0.50; 95% CI = 0.05, 5.17; one study, 50 participants, very low certainty of evidence) (**Figure 6**, **Table 2**). The RoB 2 assessment suggests a low risk of bias in all domains.

## DISCUSSION

### Acute diarrhoea with blood in stools

We found no studies comparing antibiotics with placebo for the treatment of diarrhoea with blood in stools. A systematic review published in 2010 [23] identified two placebo-controlled trials, but these were conducted in 1986 [24] and 1989 [25], and hence were not included in our review. The aforementioned review [23] found inconclusive evidence on whether antibiotics help reduce diarrhoea and fever duration compared to no antibiotics, as one study showed a significant reduction in diarrhoea at follow-up (RR = 0.30; 95% CI = 0.15, 0.59) [25], while another had comparable results for reduction in duration of diarrhoea between groups (MD = −0.30 days; 95% CI = −1.37, 0.77) [24].

The absence of more recent studies can be attributed to the ethical considerations of not prescribing antibiotics to individuals presenting with blood in their stools, as the deliberate prescription of a placebo would go against current recommendations and lead to a substantial risk of adverse health consequences [26]. The administration of antimicrobial therapy is of particular significance in resource-limited settings, as prolonged episodes of diarrhoea, such as dysentery, can have adverse effects on children’s nutritional status and growth [27].

In cases of dysentery, where empirical treatment is necessary, the under-prescription or cautious prescription of antibiotics represents a significant health care concern, as it can lead to a patient not receiving the required care. A study conducted in Kenya documented that roughly 25% of dysentery cases in Asembo and 75% in Kibera were left untreated [28]. Similarly, an evaluation of adherence to antibiotic usage based on the Integrated Management of Childhood Illness (IMCI) clinical algorithm in Papua New Guinea showed that 11% of children in outpatient settings did not receive antibiotics when they were deemed necessary [29]. The necessity to adhere to the IMCI algorithm not only plays a crucial role in avoiding unnecessary antibiotic usage, but also ensures that patients in need of antibiotic treatment receive it judiciously.

### Acute watery diarrhoea

Five studies compared antibiotics vs placebo for the treatment of acute watery diarrhoea in children under 10 years. The meta-analysis suggests a significant increase in clinical and parasitological cure, while the duration of diarrhoea reduced in children receiving the antibiotics when compared to placebo with nitazoxanide. In contrast, all-cause mortality and the need for intravenous fluid infusion were comparable between the two groups. Per the RoB 2 tool, the studies had an overall low risk of bias in all domains, suggesting that the trials were well-conducted and had relatively lower chances of introducing bias in our findings.

Our findings follow those of a 2014 review by Leibovici-Weissman et al. [30] which focussed on cholera (adults and children) and found that antibiotics significantly reduced the mean duration of diarrhoea (MD = −36.77 hours; 95% CI = −43.51, −30.03; 19 studies, n = 1013, moderate quality evidence). Despite an exhaustive search, we identified no other, recent reviews addressing the efficacy of antibiotics vs placebo for the treatment of acute watery or persistent diarrhoea. The significant results on the primary outcomes in our review came from the use of a single antibiotic and in a single setting and thus cannot be generalised.

Per our GRADE analysis, we downgraded the evidence as having low certainty due to the small sample size of the included studies and wide CIs, as this means that the results might change with the addition of more data or different study designs. The GRADE assessment underscores that the evidence lacks the precision or robustness required to recommend the use of antibiotics in cases of acute watery or persistent diarrhoea without blood in stools. Moreover, most of the included studies were over 10 years old, so their relevance in terms of bacterial susceptibility in the current times could be debated. Given these concerns, our findings should be approached with care and should highlight the necessity for a meticulously designed large-scale trial targeted at addressing the research question.

Several arguments are raised against the utilisation of antibiotics for acute non-bloody diarrhoea, with one of the most compelling being the recognition that acute diarrhoea is inherently a self-limiting disease. Regardless of its underlying causes, the majority of cases tend to resolve spontaneously in less than three days [31]. We should also take into account the low occurrence of treatable pathogens among the causative agents of acute diarrhoea, with viruses being predominant in most cases [32], the potential occurrence of side effects, the risk of developing resistant strains, the associated treatment costs, and the plausible adverse impact on the disease itself. Lastly, virtually all oral antimicrobials have the capacity to induce or exacerbate diarrhoea due to their impact on gut microflora [33].

Our analysis is limited by the included studies’ small sample size, with only one adequately powered RCT yielding conclusive results, albeit being focussed solely on all-cause mortality over a six-month period without exploring outcomes specific to acute diarrhoea. Apart from the study by Ahmed et al. [17], which was a multi-country trial, all other studies were conducted in Egypt and compared nitazoxanide with placebo, leaving the effects of other antibiotics on diarrhoea unexplored. The outcomes among all the studies also varied substantially and resulted in only one or two studies being included in meta-analysis of each outcome. In the aforementioned study [17], the outcome of 180-day all-cause mortality seemed unrelated to acute diarrhoea, as its effects or those of antibiotics do not remain for such long durations. Another limitation is the scarcity of data, evident in the inclusion of only five studies – an insufficient number to draw decisive conclusions.

## CONCLUSIONS

We found insufficient evidence for the use or no use of antibiotics in acute watery diarrhoea. Antimicrobial stewardship initiatives should continue to promote responsible antibiotic use to mitigate the global burden of diarrhoeal diseases while preserving antibiotic efficacy for future generations. There remains a need to conduct large-scale multi-country RCTs comparing antibiotics with placebo for acute diarrhoea and assessing its impact on important outcomes of diarrhoea duration, hospitalisation, stool frequency/volume, and mortality.

## Additional material


Online Supplementary Document
