# Supplementary material for: Antibiotics for acute watery or persistent with or without bloody diarrhoea in children: A systematic review and meta-analysis
Source: J Glob Health. 2024 Dec 6;14:04211. doi: 10.7189/jogh.14.04211 (PMC11622352; doi:10.7189/jogh.14.04211)
Supplement: Online Supplementary Document [file jogh-14-04211-s001.pdf]

Online Supplementary Document

SEARCH STRATEGY

PubMed:

Table S1: PubMed Search Strategy

Acute Watery Diarrhea	Children up to 10 years	Antibiotics
(("Dysentery"[MeSH Terms] OR "Dysentery"[MeSH Terms] OR "Dysentery"[All Fields] OR "dysenteries"[All Fields] OR "Dysentery"[MeSH Terms] OR "Dysentery"[All Fields] OR "dysenteries"[All Fields] OR ("dysentery, bacillary"[MeSH Terms] OR ("Dysentery"[All Fields] AND "bacillary"[All Fields]) OR "bacillary dysentery"[All Fields] OR "Dysentery"[MeSH Terms] OR "dysentery, bacillary"[MeSH Terms] OR "dysentery, amebic"[MeSH Terms] OR "Amoebic dysentery due to Entamoeba histolytica"[Supplementary Concept] OR "diarrhea"[MeSH Terms] OR "diarrhea, infantile"[MeSH Terms] OR ("diarrhea"[MeSH Terms] OR "diarrhea"[All Fields] OR "diarrheas"[All Fields] OR "diarrhoea"[All Fields] OR "diarrhoeas"[All Fields]) OR ("diarrhea"[MeSH Terms] OR	("infant"[MeSH Terms] OR "child, preschool"[MeSH Terms] OR "infant newborn mesh"[All Fields] OR "infant*"[All Fields] OR "toddler*"[All Fields] OR ("infant, newborn"[MeSH Terms] OR ("infant"[All Fields] AND "newborn"[All Fields]) OR "newborn infant"[All Fields] OR "baby"[All Fields] OR "infant"[MeSH Terms] OR "infant"[All Fields]) OR ("baby s"[All Fields] OR "babys"[All Fields] OR "infant"[MeSH Terms] OR "infant"[All Fields] OR "babies"[All Fields]) OR ("child, preschool"[MeSH Terms] OR ("child"[All Fields] AND "preschool"[All Fields]) OR "preschool child"[All Fields] OR "preschooler"[All Fields] OR "preschoolers"[All Fields] OR "preschool"[All Fields] OR "preschooler s"[All Fields] OR "preschools"[All Fields]) OR "newborn*"[All Fields] OR "neonate*"[All Fields] OR ("kindergarten"[All Fields] OR "kindergarteners"[All Fields] OR "kindergartens"[All Fields])	((("anti bacterial agents/administration and dosage"[MeSH Terms] OR "anti bacterial agents/therapeutic use"[MeSH Terms]) NOT "probiotic*"[All Fields]) OR ("metronidazole"[Supplementary Concept] OR "metronidazole"[All Fields] OR "metronidazol"[All Fields] OR "metronidazole"[MeSH Terms] OR "metronidazoles"[All Fields]) OR ("metronidazole"[Supplementary Concept] OR "metronidazole"[All Fields] OR "flagyl"[All Fields] OR "metronidazole"[MeSH Terms] OR "metronidazol"[All Fields] OR "metronidazoles"[All Fields]) OR ("cephalosporine"[All Fields] OR "cephalosporines"[All Fields] OR "cephalosporins"[Supplementary Concept] OR "cephalosporins"[All Fields] OR "cephalosporin"[All Fields] OR "cephalosporins"[MeSH

"diarrhea"[All Fields] OR "diarrheas"[All Fields] OR "diarrhoea"[All Fields] OR "diarrhoeas"[All Fields]) OR "Abdominal distension"[All Fields] OR "loose motion"[All Fields] OR "childhood diarrhea"[All Fields] OR "childhood diarrhoea"[All Fields]	OR "under 10*"[All Fields] OR "under 10*"[All Fields] OR "under-ten"[All Fields] OR "under-ten"[All Fields] OR "kid"[All Fields] OR "kids"[All Fields] OR "paediatr*"[All Fields] OR "pediatr*"[All Fields])	Terms]) OR ("ampicillin"[Supplementary Concept] OR "ampicillin"[All Fields] OR "ampicillin"[MeSH Terms] OR "ampicilline"[All Fields] OR "ampicillins"[All Fields]) OR ("tetracycline"[Supplementary Concept] OR "tetracycline"[All Fields] OR "tetracyclin"[All Fields] OR "tetracycline"[MeSH Terms] OR "tetracyclines"[MeSH Terms] OR "tetracyclines"[All Fields] OR "tetracyclins"[All Fields]) OR ("amdinocillin"[Supplementary Concept] OR "amdinocillin"[All Fields] OR "mecillinam"[All Fields] OR "amdinocillin"[MeSH Terms]) OR "beta-Lactamase Inhibitors"[All Fields] OR ("trimethoprim"[Supplementary Concept] OR "trimethoprim"[All Fields] OR "trimethoprim"[MeSH Terms] OR "trimethoprime"[All Fields] OR "trimethoprims"[All Fields]) OR ("chloramphenicol"[Supplementary Concept] OR "chloramphenicol"[All Fields] OR "chloramphenicol"[MeSH Terms] OR "chloramphenicols"[All Fields]) OR "Nalidixic Acid"[All Fields] OR ("fluoroquinolon"[All Fields]

		OR "fluoroquinolones"[Supplementary Concept] OR "fluoroquinolones"[All Fields] OR "fluoroquinolone"[All Fields] OR "fluoroquinolones"[MeSH Terms] OR "fluoroquinolonic"[All Fields] OR "fluoroquinolons"[All Fields]) OR ("azithromycin"[Supplementary Concept] OR "azithromycin"[All Fields] OR "azithromycin"[MeSH Terms] OR "azithromycine"[All Fields] OR "azithromycins"[All Fields]))) AND (clinicaltrial[Filter] OR clinicaltrialphasei[Filter] OR clinicaltrialphaseii[Filter] OR clinicaltrialphaseiii[Filter] OR clinicaltrialphaseiv[Filter] OR controlledclinicaltrial[Filter] OR meta-analysis[Filter] OR randomizedcontrolledtrial[Filter] OR review[Filter] OR systematicreview[Filter])

Cochrane Central Register of Controlled Trials:

Table S2: Cochrane Search Strategy

Acute Watery Diarrhea	Children up to 10 years	Antibiotics
#1 MeSH descriptor: [Diarrhea] explode all trees and with qualifier(s): [drug therapy - DT]	#7 ((Infant* OR toddler* OR baby OR babies OR preschool OR newborn* OR neonate* OR kindergarten OR under-10* OR	#14 ((Metronidazole OR Flagyl OR Cephalosporin OR Ampicillin OR tetracyclines OR Mecillinam OR "beta-

#2 MeSH descriptor: [Diarrhea, Infantile] explode all trees<br><br>#3 MeSH descriptor: [Dysentery, Amebic] explode all trees<br><br>#4 ((Diarrhea OR Diarrhoea OR “Abdominal distension” OR “loose motion” OR “childhood diarrhea” OR “childhood diarrhoea” OR “childhood diarrhea” OR “childhood diarrhoea”) OR ("dysentery" OR "dysenteries" OR ("dysenteries")):ti,ab,kw<br><br>#5 MeSH descriptor: [Dysentery, Bacillary] explode all trees<br><br>#6: #1 OR #2 OR #3 OR #4 OR #5	“under 10*” OR under-ten OR “under ten” OR kid OR kids OR paediatr* OR pediatr*)):ti,ab,kw<br><br>#8 MeSH descriptor: [Child] explode all trees<br><br>#9 MeSH descriptor: [Child, Preschool] explode all trees<br><br>#10 MeSH descriptor: [Infant] explode all trees<br><br>#11 MeSH descriptor: [Pediatrics] explode all trees<br><br>#12: #7 OR #8 OR #9 OR #10 OR #11<br><br>#13: #6 AND #12	Lactamase Inhibitors" OR Trimethoprim OR Chloramphenicol OR "Nalidixic Acid" OR Fluoroquinolones OR Azithromycin)):ti,ab,kw<br><br>#15 MeSH descriptor: [Anti-Bacterial Agents] explode all trees<br><br>#16: #14 OR #15<br><br>#17: #13 AND #16

Scopus:

Table S3: Scopus Search Strategy

Diarrhea	Children up to 10 years	Antibiotics
(TITLE-ABS-KEY ((diarrh* OR dysenter* OR "abdominal distension" OR "loose motion")))	((TITLE-ABS-KEY ((child* OR "preschool child*" OR schoolchild* OR kid OR kids OR toddler* OR infant OR infants OR "Infancy" OR "Baby" OR "Babies" OR "newborn*" OR "neonat*" OR preterm OR prematur* OR "pediatric*" OR "paediatric*" OR kinderg*)))) AND	((TITLE-ABS-KEY (("AntiBacterial agent*" OR metronidazole OR flagyl OR cephalosporin OR ampicillin OR tetracyclines OR mecillinam OR "beta-Lactamase Inhibitors" OR trimethoprim OR chloramphenicol OR "Nalidixic

	(LIMIT-TO (LANGUAGE , "English"))	Acid" OR fluoroquinolones O R azithromycin)) AND NOT TITLE-ABS-KEY (probiotic*)))

CINAHL:

Table S4: CINAHIL Search Strategy

Diarrhea	Children up to 10 years	Antibiotics
(((MH "Diarrhea") OR MH dehydration OR MH Dysentery OR Dysenter* OR “Abdominal distension” OR “loose motion”) AND (S4 OR S5 OR S6))	(Infant* OR toddler* OR baby OR babies OR preschool OR newborn OR neonate* OR kindergarten OR under-10 OR under-ten OR “under ten” OR kid OR kids OR paediatr* OR pediatr*)	((Metronidazole OR Flagyl OR Cephalosporin OR Ampicillin OR tetracyclines OR Mecillinam OR "beta-Lactamase Inhibitors" OR Trimethoprim OR Chloramphenicol OR "Nalidixic Acid" OR Fluoroquinolones OR Azithromycin))

Clinicaltrials.gov:

Table S5: Clinicaltrials.gov Search Strategy

Acute Watery Diarrhea	Children up to 10 years	Antibiotics
(("Dysentery" OR "Dysentery" OR "Dysentery" OR "dysenteries" OR "Dysentery" OR "Dysentery" OR "dysenteries" OR ("dysentery, bacillary" OR ("Dysentery" AND "bacillary") OR "bacillary dysentery" OR "Dysentery" OR "dysentery, bacillary" OR "dysentery, amebic" OR	("infant" OR "child, preschool" OR "infant newborn mesh" OR "infant*" OR "toddler*" OR ("infant, newborn" OR ("infant" AND "newborn") OR "newborn infant" OR "baby" OR "infant" OR "infant") OR ("baby s" OR "babys" OR "infant" OR "infant" OR "babies") OR ("child, preschool" OR ("child" AND "preschool") OR	((("anti bacterial agents/administration and dosage"OR "anti bacterial agents/therapeutic use") NOT "probiotic*") OR ("metronidazole" OR "metronidazole" OR "metronidazol" OR "metronidazole" OR

"Amoebic dysentery due to Entamoeba histolytica" OR "diarrhea" OR "diarrhea, infantile" OR ("diarrhea" OR "diarrhea" OR "diarrheas" OR "diarrhoea" OR "diarrhoeas") OR ("diarrhea" OR "diarrhea" OR "diarrheas" OR "diarrhoea" OR "diarrhoeas") OR "Abdominal distension" OR "loose motion" OR "childhood diarrhea" OR "childhood diarrhoea"	"preschool child" OR "preschooler" OR "preschoolers" OR "preschool" OR "preschooler s" OR "preschools") OR "newborn*" OR "neonate*" OR ("kindergarten" OR "kindergarteners" OR "kindergartens") OR "under 10*" OR "under 10*" OR "under-ten" OR "under-ten" OR "kid" OR "kids" OR "paediatr*" OR "pediatr*")	"metronidazoles") OR ("metronidazole" OR "metronidazole" OR "flagyl" OR "metronidazole" OR "metronidazol" OR "metronidazoles") OR ("cephalosporine" OR "cephalosporines" OR "cephalosporins" OR "cephalosporins" OR "cephalosporin" OR "cephalosporins") OR ("ampicillin" OR "ampicillin" OR "ampicillin" OR "ampicilline" OR "ampicillins") OR ("tetracycline" OR "tetracycline" OR "tetracyclin" OR "tetracycline" OR "tetracyclines" OR "tetracyclines" OR "tetracyclins") OR ("amdinocillin" OR "amdinocillin"[OR "mecillinam" OR "amdinocillin") OR "beta-Lactamase Inhibitors" OR ("trimethoprim" OR "trimethoprim" OR "trimethoprim" OR "trimethoprime" OR "trimethoprims") OR ("chloramphenicol" OR "chloramphenicol" OR "chloramphenicol" OR "chloramphenicols")

		OR "Nalidixic Acid" OR ("fluoroquinolon" OR "fluoroquinolones" OR "fluoroquinolones" OR "fluoroquinolone" OR "fluoroquinolones" OR "fluoroquinolonic" OR "fluoroquinolons") OR ("azithromycin" OR "azithromycin" OR "azithromycin" OR "azithromycine" OR "azithromycin s"))) AND (clinicaltrial OR clinicaltrialphasei OR clinicaltrialphaseii OR clinicaltrialphaseiii OR clinicaltrialphaseiv OR controlledclinicaltrial OR meta-analysis OR randomizedcontrolledtrial OR review OR systematicreview)
